# Huaier suppresses pancreatic cancer progression *via* activating cell autophagy induced ferroptosis

**DOI:** 10.3389/fonc.2022.960858

**Published:** 2022-09-30

**Authors:** Zeen Zhu, Xueni Wang, Wunai Zhang, Mengyuan Gong, Simei Zhang, Bao Yang, Bolun Qu, Zheng Wu, Qingyong Ma, Zheng Wang, Weikun Qian

**Affiliations:** ^1^ Department of Hepatobiliary Surgery, The First Affiliated Hospital of Xi’an Jiaotong University, Xi’an, China; ^2^ Pancreatic Disease Center of Xi’an Jiaotong University, Xi’an, China

**Keywords:** Pancreatic cancer, Huaier, ferroptosis, autophagy, reactive oxygen species

## Abstract

**Purpose:**

The anti-tumour effect of Huaier has been demonstrated in a variety of tumours. Ferroptosis is a newly identified type of programmed cell death accompanied by the accumulation of reactive oxygen species (ROS) and iron in cells and plays a key role in the therapeutic process against malignant tumours. We aimed to explore the potential therapeutic role of Huaier in pancreatic cancer and uncover the relationship between Huaier and ferroptosis.

**Methods:**

CCK8 and colony formation assays were used to determine the proliferation of pancreatic cancer cells (PCs). The levels of cellular ROS were analysed by a fluorescence probe, and the accumulation of cellular iron was showed by Prussian blue staining. The autophagosomes and mitochondrial morphology were characterised by transmission electron microscopy (TEM). The levels of intracellular glutathione (GSH) and lipid peroxidation were measured by the corresponding kits.

**Results:**

The growth inhibitory effect of Huaier on PCs was concentration- and time-dependent, but this effect was significantly attenuated by ferroptosis inhibitors. In addition, Huaier effectively inhibited the GSH–GPX4 antioxidation system and resulted in the massive accumulation of ROS in PCs As shown by TEM, Huaier-treated PCs exhibited a decrease in mitochondrial cristae and a smaller mitochondrion, accompanied by an increase in autophagosomes. Indeed, we found that autophagy can induce ferroptosis in PCs and that Huaier-induced ferroptosis can be suppressed by the autophagosome inhibitor, Wortmannin.

**Conclusion:**

Huaier can activate ferroptosis by inducing autophagy in PCs.

## Introduction

Pancreatic ductal adenocarcinoma (PDAC) is a common digestive cancer with a high mortality rate (1). Due to the unique anatomical location of pancreas, most patients with PDAC are diagnosed at an advanced stage and lose the opportunity for surgical treatment (2). According to an epidemiological study, the global morbidity and mortality of pancreatic cancer increased by 253,000 and 245,000, respectively, from 1990 to 2017 (3). These figures indicate that the incidence and mortality of PDAC are gradually increasing. Currently, surgical resection combined with chemotherapy and radiotherapy is still the main methods for the clinical treatment of PDAC (4), but due to the complex tumour microenvironment, neither radiotherapy nor chemotherapy works well (5). Overall, the 5-year survival rate does not exceed 10% because of various limitations, especially the resistance to chemotherapy (6). Therefore, research into new effective agents to avoid chemotherapy resistance has become an important undertaking to improve the clinical efficacy of PDAC treatments.

Recently, potential clinical anti-tumour agents from natural sources have attracted increasing attention. The anti-tumour properties of various herbal medicines have been discovered. Huaier is a fungus that grows on acacia trees and was approved by the State Food and Drug Administration (SFDA) of China for clinical use (No. Z20000109) (7). Recent studies have shown that Huaier has anti-tumour effects in many malignant tumours, such as lung cancer (8), prostate cancer (9), breast cancer (10) and pancreatic cancer (11). However, the anti-tumour mechanisms of Huaier are still not fully understood.

In 2012, Dixon et al. discovered ferroptosis, a new form of cell death, which was different from necrosis, apoptosis, and autophagy (12). Ferroptosis involves alterations in intracellular iron homeostasis and iron metabolism, the accumulation of reactive oxygen species (ROS) and the specific manifestation of lipid peroxidation. Morphologically, TEM reveals that ferroptotic cells exhibit a state with accumulated chromosomes, reduced sizes of mitochondria, increased density of mitochondrial membranes and reduction or vanishment of cristae (13). Erastin and RSL3 are two classic drugs capable of inducing ferroptosis. Among them, erastin can suppress SLC7A11 (a highly specific transport component of cysteine and glutamate, so called system Xc (-), the critical cell antioxidant system and main mediator of cell ferroptosis) and lead to glutathione (GSH) depletion. GSH exhaustion leads to the inactivation of GSH peroxidase 4 (GPX4), which is a key component that inhibits lipid peroxidation, but RSL3 directly inactivates GPX4, thus inducing ferroptosis (14). Interestingly, arsenic was reported to induce oxidative stress, and ROS caused the peroxidation of cell membrane lipids, resulting in ferroptosis (15); therefore, there are also other drugs that can induce ferroptosis, and the underlying mechanisms need to be clarified. For example, autophagy is a ubiquitous biological process in mammalian cells in which autophagosomes fuse with lysosomes to form degradative autolysosomes (16), and it has been reported that autophagy can promote ferroptosis *via* autophagy related (ATG) genes mediated regulated cell death (RCD) (17, 18).

In our study, we found that Huaier can induce cell death with morphologies similar to ferroptosis, cause the accumulation of ROS and induce autophagy in PCs. Huaier is a complex natural compound, and the main objectives of our study were to determine whether Huaier can induce ferroptosis and to uncover the relationship between autophagy and Huaier-induced ferroptosis.

## Methods and materials

### Cell culture and reagents

The human pancreatic cancer cell lines Panc-1 and MiaPaCa-2 were obtained from the Cell Bank of the Chinese Academy of Sciences (Shanghai, China) and were cultured in Dulbecco’s modified Eagle’s medium (DMEM; Gibco; USA) supplemented with 10% foetal bovine serum (FBS) and 1% penicillin and streptomycin. A humidified atmosphere at 37°C with 5% CO2 was utilised for culture. Huaier extract was provided by Gaitianli Medicine Co., Ltd. (Qidong, Jiangsu, China). All protocols were approved by the Ethical Committee of the First Affiliated Hospital of Xi’an Jiaotong University, Xi’an, China.

### Cell viability assay

Panc-1 and MiaPaCa-2 cells were seeded into 96-well plates at 3000 cells per well. After an overnight incubation, different concentrations of Huaier and other treatment strategies (for details, see the corresponding results section) were added, and the cells were incubated for another 24 h, 48 h, 72 h, and 96 h. After incubation, the culture medium in the 96-well plates was removed, and 100 μL DMEM and 10 μL cell counting kit 8 (CCK8) were added to each well and incubated for another 3 h at 37°C with 5% CO2. The absorbance was detected at 450 nm by using a multifunction microplate reader (POLARstar OPTIMA; BMG, Offenburg, Germany).

### Colony formation assay

The Panc-1 and MiaPaCa-2 cells were plated in 6 cm Petri dishes for 72 h (1000 per dish) to induce colony formation and were treated with or without Huaier and other treatment strategies (for details, see the corresponding results section) for 2 weeks. Afterward, each dish was washed with phosphate-buffered saline (PBS) three times, fixed with 4% paraformaldehyde, stained with crystal violet for 20 min and photographed.

### Detection of intracellular ROS

Panc-1 and MiaPaCa-2 cells were plated into six-well plates, and each dish contained at least 10000 cells that were treated with different concentrations of Huaier for 24 h. Then, the medium was removed from the dishes, and the cells were washed three times with PBS. A Reactive Oxygen Species Assay Kit (Biyuntian Institute of Biotechnology, China) was used to measure the content of intracellular ROS. The quantification of ROS production was performed by using a ZEISS fluorescence microscope and ImageJ software (version 1.52a, National Institutes of Health, Bethesda, MD, USA).

### Western blot analysis

Panc-1 and MiaPaCa-2 cells were seeded in 6 cm dishes and treated with or without Huaier and other treatment strategies (for details, see the corresponding results section) until the density of the cells was 70%-80% for 24 h. RIPA lysis buffer (400 µl) supplemented with 4 µl protease and 8 µl phosphatase inhibitors was added to each dish. Then, the samples were centrifuged at 12000 × g for 20 min at 4°C, and the supernatant was collected and boiled for 7 min. The protein samples were separated on a 10% or 15% SDS gel and transferred to polyvinylidene fluoride (PVDF) membranes. After the PVDF membranes were blocked for 1 hour with blocking solution, primary antibodies against P62 (Proteintech, China), ATG5 (Proteintech, China), LC3 (Proteintech, China), SLC7A11 (Beyotime, China), GPX4 (Abcam, USA), COX2 (CST, USA), P-ULK (CST, USA), P-ERK (CST, USA), BECLIN (CST, USA), P-EGFR (CST, USA), and ACTIN (CST, USA) were used for an overnight incubation at 4°C. Then, the membranes were washed with PBST three times and were incubated with goat anti-rabbit or goat anti-mouse secondary antibodies (CST, USA) for 1 h, followed by three washes with PBST. The Molecular Imager ChemiDoc XRS System (Bio–Rad, Hercules, CA, USA) was used to detect the membranes.

### Cell transfection

For autophagy detection, adenovirus (ad) fluorescent mRFP-GFP-LC3 was purchased from HanBio Technology Co., Ltd., China. Cells were transfected according to the manufacturer’s recommendation. The analysis of autophagic flux was performed using a ZEISS fluorescence microscope and ImageJ software (version 1.52a, National Institutes of Health, Bethesda, MD, USA).

### Immunofluorescence analysis

Pancreatic cancer cells were grown in 6-well culture plates, and after 24 h, the cells were treated with or without Huaier. After 24 h, the cells were washed with PBS, fixed for 10 min at room temperature with 4% paraformaldehyde, and permeabilized for 15 min with 0.5% Triton X-100 in PBS. Then, the cells were blocked with 5% normal serum in PBS for 1 h. Next, the cells were incubated at 4°C with the desired primary antibody overnight. Images were analysed using a ZEISS fluorescence microscope and ImageJ software (version 1.52a, National Institutes of Health, Bethesda, MD, USA).

### GSH and lipid peroxidation assays

Cell-lysate GSH and malondialdehyde (MDA) concentrations were analysed with a GSH assay kit (Beyotime, China) and lipid peroxidation assay kit (Beyotime, China), respectively, according to the manufacturers’ protocols.

### Transmission electron microscopy (TEM)

Cells were digested in 0.25% trypsin and fixed with 2.5% glutaraldehyde for 24 hours. The samples were postfixed in 2% aqueous osmium tetroxide, dehydrated in graded ethanol solutions (70%–100%) with propylene oxide, embedded in Epon 812 (Merck), and incubated for 48 h at 60°C. Semithin sections (1.0 μm) were cut and stained with toluidine blue. Ultrathin sections (80 nm) were collected onto 200-mesh copper grids and stained with uranyl acetate and lead citrate prior a TEM examination with an H-7700 microscope (Hitachi, Tokyo, Japan).

### TCGA data portal

The analyses are based upon data generated by TCGA, which is managed by the NCI and NHGRI. Specifically, the projects on PDAC, which is usually called PAAD, in the TCGA database (TCGA-PAAD; https://portal.gdc.cancer.gov/projects/TCGA-PAAD) were included. The access to controlled datasets was approved by NIH (project ID 20041).

### *In vivo* study

A nude mouse-based pancreatic subcutaneous tumour model of pancreatic cancer was established in this study. All experimental protocols were approved by the Ethical Committee of the First Affiliated Hospital of Xi’an Jiaotong University, Xi’an, China. When nude mice were 6-7 weeks of age, 1×10^6 Panc-1 cells were resuspended in a 1:3 (v/v) mixture of culture medium, and Matrigel (BD Biosciences, San Jose, CA, USA) was injected into the dorsal thigh root subcutaneously. On the 7^th^ day after inoculation, the nude mice were randomly divided into the following two groups (the control group (saline 100 µL/day, gavage) and Huaier group (2 g/kg/day, gavage); five mice per group) and three groups (the control group (saline 100 µL/day, gavage), Huaier group (2 g/kg/day, gavage), and Huaier plus ferrostatin-1 group (100 mg/kg/day, gavage); five mice per group). Notably, for choosing the propriate dose of Huaier, we first referred to the dose of commercial available Huaier for clinical use (7.92 g/day for a 60 kg person which means 0.13 g/kg/day); then according to the Reagan-Shaw method for dose translation from animal to human (19) that mice have 12.3 times the equivalent dose to humans (0.13 g/kg/day for human = 1.6 g/kg/day for mice); finally, excluding the approximate 20% of weight loss in drug preparation and gavage, we adopted 2 g/kg/day for *in vivo* Huaier treatment. And this was consisting with our previous reported 50 mg/0.1ml/day Huaier for each adult (about 25 g) nude mice (11). At the end of the 6^th^ week of intervention, the nude mice were sacrificed, and the tumour volume and/or tumour weight were examined. The subcutaneous tumours obtained from the animal model were preserved in 4% buffered formalin and embedded in paraffin. Haematoxylin & Eosin (H&E) staining and immunohistochemical (IHC) staining-based pathological analysis were performed by using a commercial kit according to the manufacturer’s protocol. The study was conducted in accordance to the Declaration of Helsinki, and all protocols were approved by the Ethical Committee of the First Affiliated Hospital of Xi’an Jiaotong University, Xi’an, China.

### Statistical analysis

The data are presented as the mean ± SD. Student’s t test and one-way ANOVA were used to verify the comparison between two groups by using the GraphPad Prism software package (GraphPad Prism version 6.0; La Jolla, CA, USA). P<0.05 was considered to indicate a significant difference.

## Results

### Huaier inhibited the proliferation of pancreatic cancer cells *in vitro* and tumour growth *in vivo*

**Figure 1:**
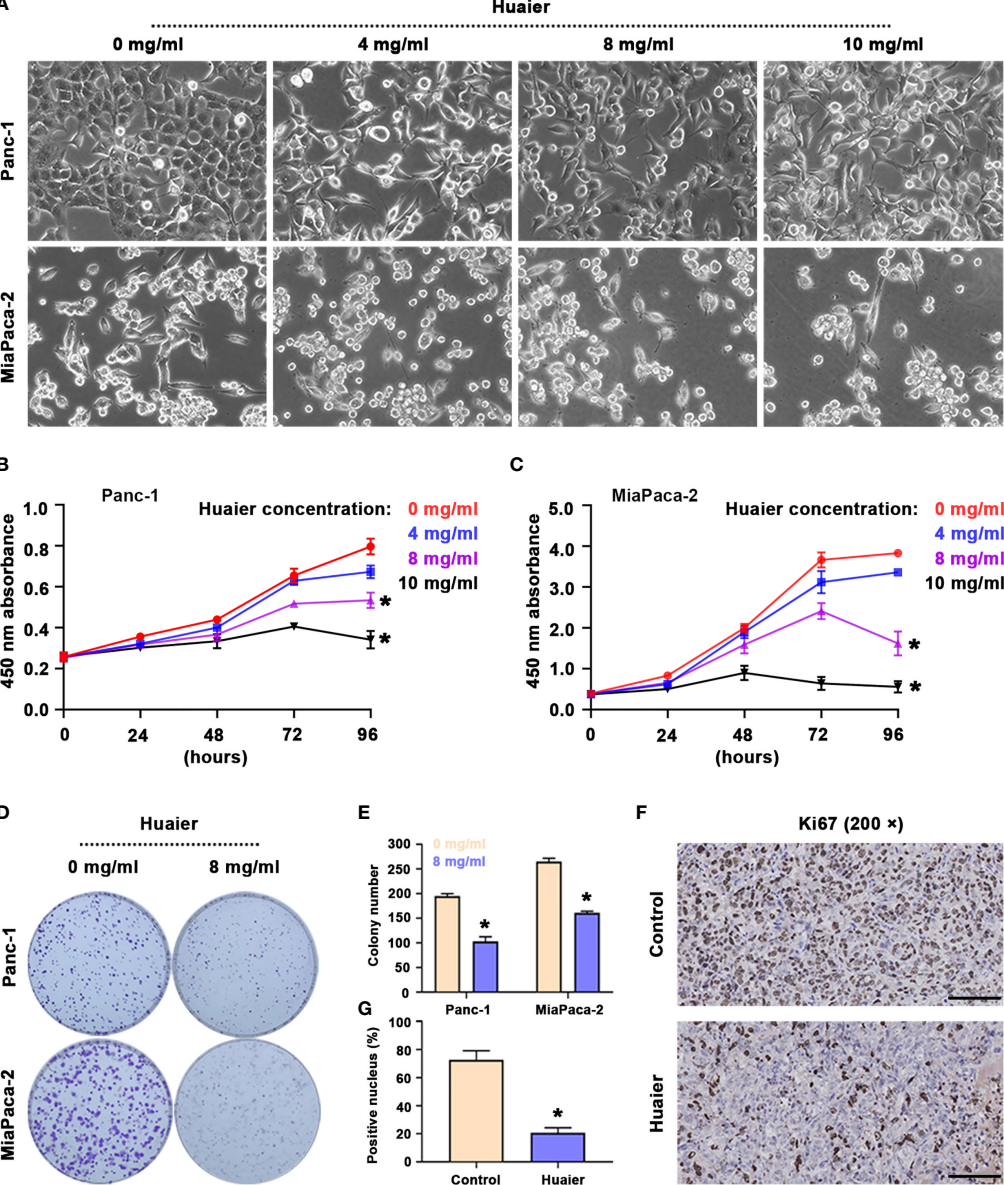
Huaier inhibited pancreatic cancer cell proliferation *in vitro* and tumour growth *in vivo*. **(A)** Bright field images showing the cell morphology of Panc-1 and MiaPaCa-2 cells after treatment with various concentrations of Huaier. **(B, C)** CCK8 assay showing the viability of Panc-1 and MiaPaCa-2 cells after treatment with various concentrations of Huaier for 24 h, 48 h, 72 h, and 96 h. **(D, E)** Colony formation assay and its statistical analysis showing the cell proliferation of Panc-1 and MiaPaCa-2 cells after Huaier treatments for 2 weeks. **(F, G)** IHC staining of Ki67 and its statistical analysis showing the proliferation status of pancreatic cancer tumours in the control and Huaier groups. *P < 0.05, scale bar = 100 μm.

The results suggest that following the intervention of Huaier for 48 h, the morphology of Panc-1 and MiaPaCa-2 pancreatic cancer cells was significantly altered (**Figure 1A**), as reflected by the stretching observed in the cell morphology, the broken intercellular connections, and the loose cell arrangement, which reminded us that Huaier may influence biological behaviours, such as cell death, cell proliferation and cell migration. To research the precise effect of Huaier, cell proliferation/death were examined, as they are the most common phenotypes of cancer cells; therefore, we treated cells with different concentrations of Huaier for 24 h, 48 h, 72 h, and 96 h, and the cell viability inhibition was determined by a CCK8 assay. The results revealed that Huaier inhibited cell growth in a concentration-dependent manner in Panc-1 and MiaPaCa-2 cells (**Figures 1B, C**). In addition, the colony formation assay also confirmed the inhibitory role of Huaier on the proliferation of Panc-1 and MiaPaCa-2 cells (**Figures 1D, E**). Moreover, at the *in vivo* level, the proportion of Ki67-positive areas was significantly decreased compared with that observed in the control group (**Figures 1F, G**). Besides, LDH activity measurement showed Huaier increased the activity of LDH in a dose-dependent manner (**Table S1**), which means Huaier mediated cell proliferation inhibition may be induced by cell death. To sun up, these results indicated that Huaier can suppress the proliferation of pancreatic cancer cell *in vitro* and pancreatic tumour growth *in vivo*.

### Huaier inhibited the proliferation of pancreatic cancer cells *in vitro* by inducing ferroptosis

**Figure 2:**
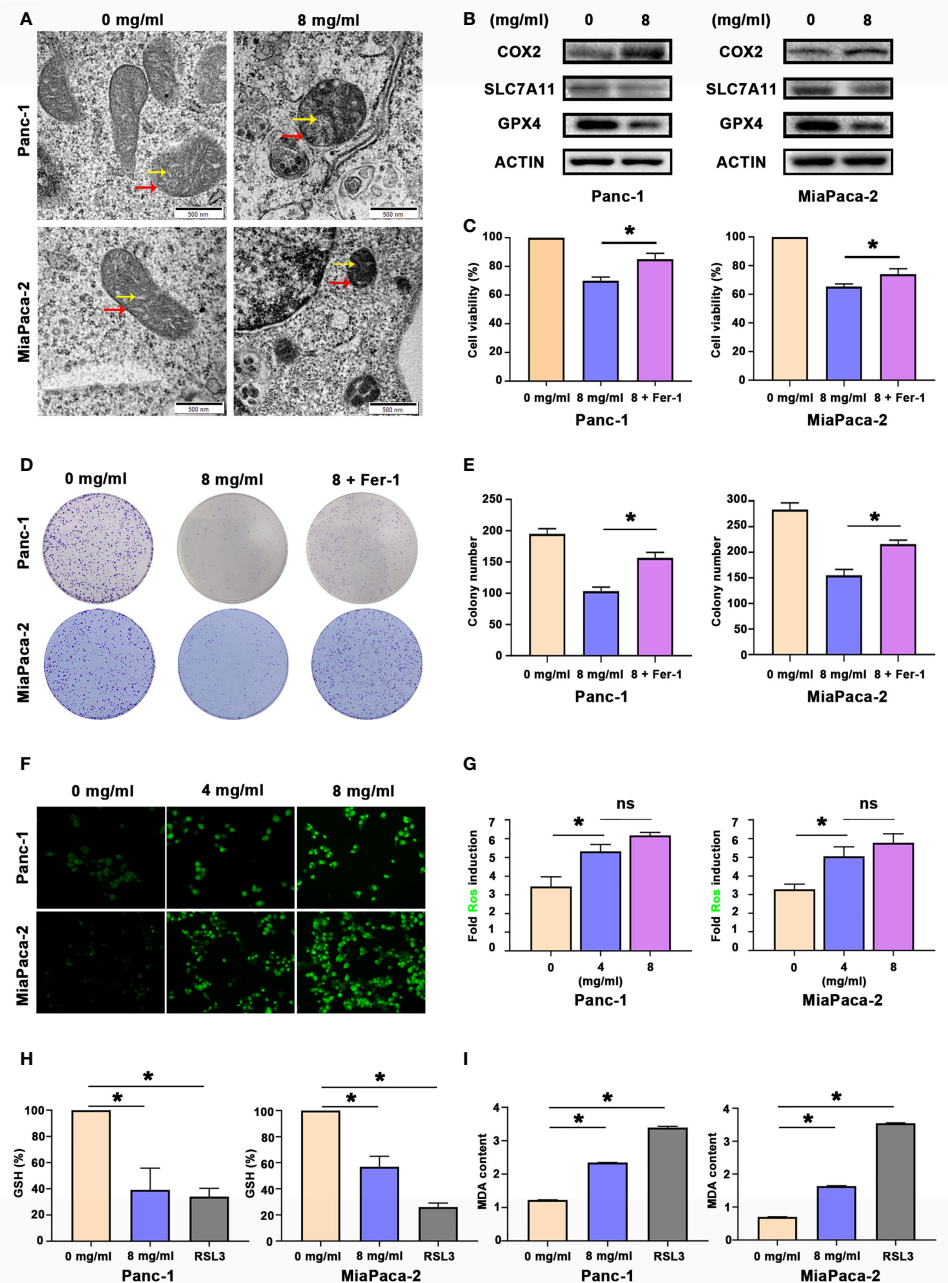
Huaier inhibited pancreatic cancer cell proliferation *in vitro* by inducing ferroptosis. **(A)** TEM images showing the mitochondria in Panc-1 and MiaPaCa-2 cells after the Huaier treatment (the yellow arrow reflects cristae, and the red arrow reflects the outer membrane). **(B)** Western blotting showing the protein levels of COX2, SLC7A11 and GPX4 in Panc-1 and MiaPaCa-2 cells after the Huaier treatment. **(C)** CCK8 assay showing the viability of Panc-1 and MiaPaCa-2 cells after the control, Huaier and Huaier plus ferrostain-1 (Fer-1) treatments for 96 h. **(D, E)** Colony formation assay and its statistical analysis showing the cell proliferation of Panc-1 and MiaPaCa-2 cells after the control, Huaier and Huaier plus Fer-1 treatment for 2 weeks. **(F, G)** Immunofluorescence-based DCFH-DA staining and statistical analysis showing the intracellular ROS levels in Panc-1 and MiaPaCa-2 cells after treatment with various concentrations of Huaier. **(H, I)** The measurement of GSH and MDA concentrations in Panc-1 and MiaPaCa-2 cells after the control, Huaier and RSL3 treatments. ns P > 0.05, *P < 0.05, scale bar = 500 nm.

Since Huaier can inhibit the proliferation of pancreatic cell and tumour growth, our next step was to explain how Huaier causes death of pancreatic cancer cells. Panc-1 and MiaPaCa-2 were treated with control medium and Huaier for 24 h, and the cells were processed for TEM analyses. An interesting phenomenon was observed (**Figure 2A**). The red arrows indicated that the mitochondrial volume decreased and the membrane density increased, while the yellow arrows indicated that mitochondrial cristae fused and even disappeared. These phenomena were in accordance with previous articles that reported the description of ferroptosis, so we speculate that Huaier can cause ferroptosis in pancreatic cancer cells. To explore whether Huaier induced ferroptosis *in vitro*, we first measured the ferroptosis-related proteins COX2 (a critical enzyme responsible for lipid peroxidation, the intrinsic characteristic of ferroptosis), GPX4 and SLC7A11 in Panc-1 and MiaPaCa-2 cells (**Figure 2B**). Compared with the control group, GPX4 and SLC7A11 expression was obviously decreased, and COX2 expression was significantly increased in the Huaier group. In addition, compared with the control group, Prussian blue staining showed heavy cellular iron accumulation in the Huaier group (**Figure S1A**). In addition, CCK8 and colony formation assays showed that ferrostatin-1 (Fer-1), a selective ferroptosis inhibitor, rescued the cell death induced by Huaier (**Figures 2C–E**). Moreover, we decided to explore whether Huaier can produce ROS because ROS generation is an essential process during ferroptosis. The results revealed that the fluorescence intensity of ROS (bright green) was enhanced by Huaier compared with that of the control group, but there was no significant difference between the groups treated with different concentrations of Huaier (4 mg/ml, 8 mg/ml) (**Figures 2F, G**). Furthermore, we applied the classic ferroptosis inducer RSL3 to verify whether Huaier could induce ferroptosis-like cell death *in vitro*. The oxidative stress levels were evaluated by the examination of GSH and MDA (two markers of lipid peroxidation that act as the terminal event of ferroptosis) in pancreatic cancer cells. A significant reduction in GSH and an increasing in MDA after Huaier or RSL3 treatment was observed compared with that of the control group (**Figures 2H, I**). These results indicate that Huaier can induce ferroptosis in pancreatic cancer cells.

### Huaier inhibited pancreatic cancer tumour growth *in vivo* by inducing ferroptosis

**Figure 3:**
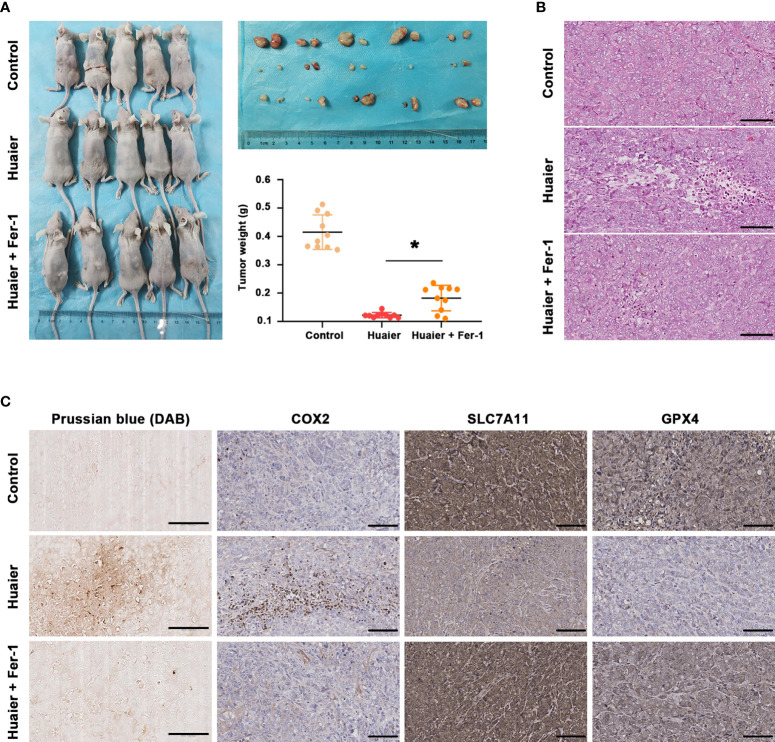
Huaier inhibited pancreatic cancer tumour growth *in vivo* by inducing ferroptosis. **(A)** Macroscopic images of pancreatic cancer tumours in the control, Huaier and Huaier plus ferrostain-1 (Fer-1) groups and statistical graph of their tumour weights. **(B)** H&E staining showed the micropathological morphology of pancreatic cancer tumours in the control, Huaier and Huaier plus Fer-1 groups. **(C)** Prussian blue staining and IHC staining of COX2, SLC7A11 and GPX4 in pancreatic cancer tumours in the control, Huaier and Huaier plus Fer-1 groups. *P < 0.05, scale bar = 100 μm.

By using a Panc-1-cell-based subcutaneous transplanted tumour model, we aimed to verify the effect of Huaier on tumour growth and ferroptosis *in vivo*. After treatment with Huaier, normal saline or Huaier plus Fer-1 for 6 weeks, all mice were sacrificed, and the tumours were collected. We found that Huaier markedly suppressed tumour growth, and a robust decrease in tumour weight was observed in the Huaier group compared with the control group, but this phenomenon was reversed by Fer-1 (**Figure 3A**). In addition, H&E staining showed a widespread necrotic area in the Huaier group compared with the control group, and this change was reversed by Fer-1 (**Figure 3B**). Next, to determine whether the necrosis induced by Huaier was ferroptosis, Prussian blue staining was adopted, and the results showed heavy cellular iron accumulation in the Huaier group (**Figure 3C**). Indeed, IHC staining also showed that COX2 was higher, while the ferroptosis proteins SLC7A11 and GPX4 were lower in the subcutaneous tumour specimens of the Huaier-treated group compared to those of the control group. However, all these changes were reversed by Fer-1 (**Figure 3C**). In brief, this evidence confirmed that Huaier inhibited tumour growth *in vivo* by inducing ferroptosis.

### Huaier increased autophagosome in pancreatic cancer

**Figure 4:**
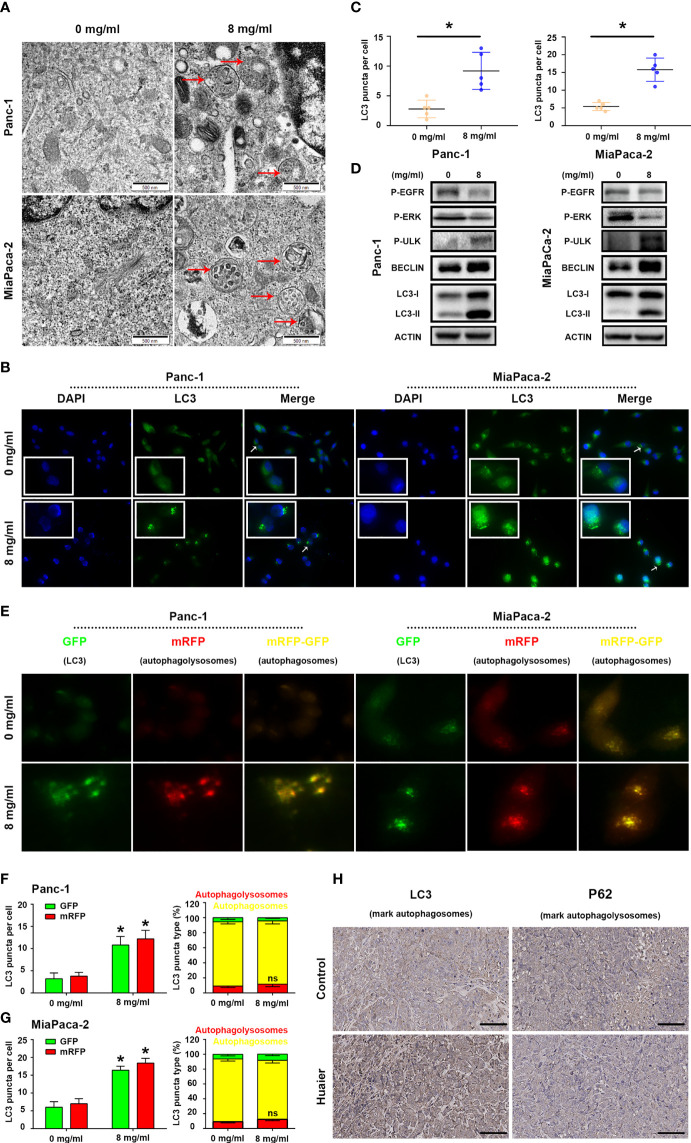
Huaier induced autophagy in pancreatic cancer. **(A)** TEM images showing autophagosomes (red arrows) in the Panc-1 and MiaPaCa-2 cells after the Huaier treatment. **(B, C)** Immunofluorescence staining of LC3 puncta (white arrows) and statistical analysis of the Panc-1 and MiaPaCa-2 cells after Huaier treatment. **(D)** Western blotting showing the protein levels of P‐EGFR, P-ERK, P-ULK, BECLIN and LC3 in Panc-1 and MiaPaCa-2 cells after Huaier treatment. **(E–G)** ad-mRFP-GFP-LC3-based fluorescence images of autophagosomes (yellow) and autophagolysosomes (red) and their statistical analysis in Panc-1 and MiaPaCa-2 cells after Huaier treatment. **(H)** IHC staining of LC3 and P62 showing the autophagy status of pancreatic cancer tumours in the control and Huaier groups. *P < 0.05, scale bar = 100 μm.

While using electron microscopy to photography the Huaier-treated pancreatic cancer cells to evaluate ferroptosis, we occasionally found that the number of autophagosomes in the Huaier group was significantly higher than that in the WT group (**Figure 4A**), so we wondered whether Huaier also regulates autophagy and specific stage in pancreatic cancer cells. Firstly, we performed immunofluorescence staining with LC3 in pancreatic cancer cells to mark autophagy spots, and the results revealed that Huaier treatment significantly up-regulated the expression of the autophagy marker LC3 (**Figures 4B, C**). Consistent with this, a western blotting assay (**Figure 4D**) also showed that the autophagy biomarkers LC3-II and BECLIN protein increased upon treating the pancreatic cancer cells with Huaier; besides, we also found that Huaier decreased the levels of P-EGFR and P-ERK and had the opposite effect on P-ULK/Ser317 (**Figure 4D**), which suggested the autophagy induced by Huaier may be a multiple process, such as inhibition of EGFR/ERK pathway (a demonstrated autophagy inhibition pathway that is induced by Huaier in other tumours (20)) and activation of AMPK pathway.

Indeed, to determine the main stage (namely, the mechanisms) of autophagy that Huaier influenced in pancreatic cancer cells, we detected autophagic flux by using ad-mRFP-GFP-LC3, and the results showed that Huaier can increased the number of autophagosomes (yellow-labelled LC3); however, it has no effect on “smoothing” the process of autophagic flux (**Figures 4E–G**, no difference between on the level of red-labelled LC3 reflect autophagolysosome in the control and Huaier groups), which reminds us Huaier-induced increase in autophagosomes may be partly due to accumulation effect. In other words, Huaier mainly influenced early autophagy rather than late autophagy. Consistent with this, the IHC assay showed high levels of the early autophagy markers LC3 (**Figure 4H**) and BECLIN/ATG5 (data not shown) in the Huaier-treated pancreatic tumour group, but the level of late autophagy markers P62 did not differ between the control and Huaier groups (**Figure 4H**). Besides, in view of the fact mentioned in **Figure 4D** (LC3II, BECLIN, ULK was the marker of the formation of autophagy in autophagosome stage), we can still believe that apart from the accumulation effect, Huaier also promoted the production of autophagosome. Taken together, both *in vitro* and *in vivo* experimental results demonstrate that Huaier can increased autophagosome in pancreatic cancer.

### Autophagy promoted ferroptosis in pancreatic cancer

**Figure 5:**
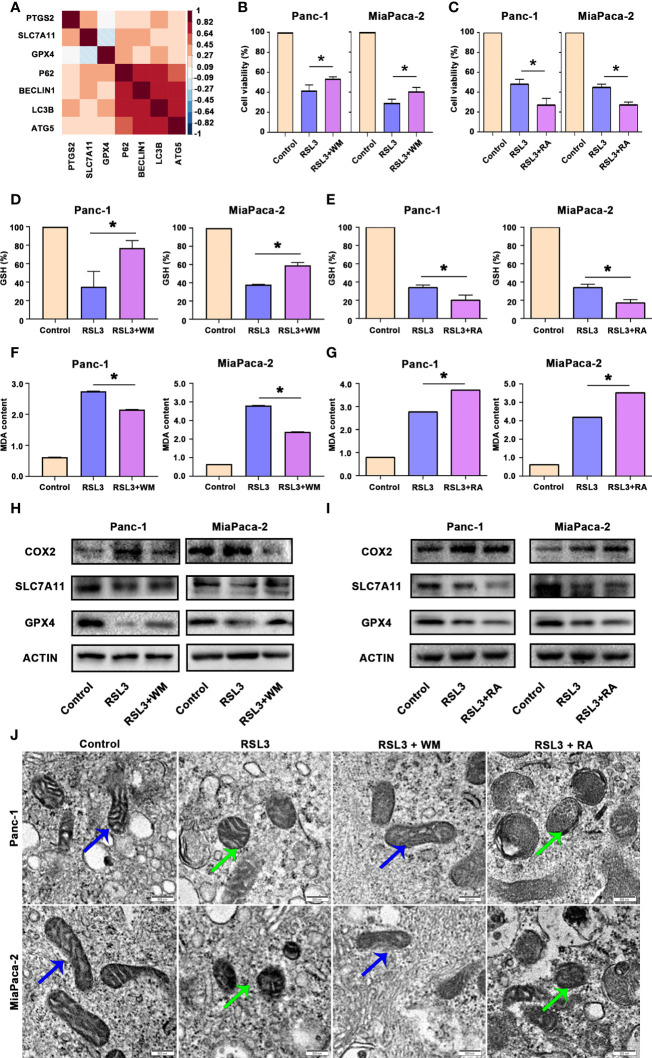
Autophagy promoted ferroptosis in pancreatic cancer. **(A)** Hierarchical clustering results in the TCGA-PAAD cohort of 7 genes involved in ferroptosis and autophagy. Correlation map reporting Spearman correlation values for each comparison. The bar on the left of the map indicates the colour legend of the Pearson correlation values calculated for each pair of genes in the matrix. **(B)** CCK8 assay showing the viability of Panc-1 and MiaPaCa-2 cells after the control, RSL3 and RSL3 plus wortmannin (WM) treatments for 96 h. **(C)** CCK8 assay showing the viability of Panc-1 and MiaPaCa-2 cells after the control, RSL3 and RSL3 plus rapamycin (RA) treatments for 96 h. **(D, F)** The measurement of GSH and MDA concentrations in Panc-1 and MiaPaCa-2 cells after the control, RSL3 and RSL3 plus WM treatments. **(E, G)** The measurement of GSH and MDA concentrations in Panc-1 and MiaPaCa-2 cells after the control, RSL3 and RSL3 plus RA treatments. **(H)** Western blotting showing the protein levels of COX2, GPX4 and SLC7A11 in RSL3 in Panc-1 and MiaPaCa-2 cells after the control, RSL3 and RSL3 plus WM treatments. **(I)** Western blotting showing the protein levels of COX2, GPX4 and SLC7A11 in RSL3 in Panc-1 and MiaPaCa-2 cells after the control, RSL3 and RSL3 plus RA treatments. **(J)** TEM images showing mitochondria (blue arrows reflect normal like and green arrows reflect ferroptosis like) in Panc-1 and MiaPaCa-2 cells after the control, RSL3, RSL3 plus WM and RSL3 plus RA treatments. *P < 0.05, scale bar = 500 nm.

There is a large amount of evidence that autophagy can promote ferroptosis (21). By analysing the TCGA dataset of pancreatic cancer (TCGA PAAD), we found that autophagy biomarkers, such as LC3B-II, were correlated with ferroptosis biomarkers, such as GPX4 (**Figure 5A**). Hence, we treated pancreatic cancer cells with RSL3 (a well-known ferroptosis inducer that inhibits GPX4) in the presence of Wortmannin (WM, a well-known autophagosome inhibitor that supresses autophagosome) or Rapamycin (RA, a well-known autophagy activator that inhibits mTOR). The results suggested that RSL3 suppressed the viability of Panc-1 and MiaPaCa-2 cells, whereas the autophagosome inhibitor WM could rescue the growth inhibition induced by RSL3 (**Figure 5B**); however, the autophagy activator RA strengthened the growth inhibition effect of RSL3 in pancreatic cancer cells (**Figure 5C**), which reminds us that there may be a relationship between autophagy and ferroptosis in pancreatic cancer. To further confirm this potential link, we found that pretreating pancreatic cancer cells with WM impaired the reduction in GSH and that the overload of MDA under RSL3 exposure was alleviated (**Figures 5D, F**); however, after a pretreatment with RA, the influence of GSH and MDA under RSL3 exposure was exacerbated (**Figures 5E, G**). Moreover, we measured the levels of COX2, SLC7A11 and GPX4 proteins in these cell groups, and the results showed that the RSL3-induced downregulation of GPX4 and SLC7A11 and upregulation of COX2 were reversed by WM and worsened by RA (**Figures 5H, I**). Additionally, electron microscopy (**Figure 5J**) also showed that the suppression of autophagosome inhibited ferroptosis; however, the promotion of autophagy had the opposite effect. In addition, Prussian blue staining showed that intracellular iron deposits were increased in RSL3-treated pancreatic cancer cells, and this effect could be alleviated by WM but was accentuated by RA (**Figure S1B**). Together, these data demonstrate that autophagy is an active regulator of ferroptosis in pancreatic cancer cells.

### Huaier facilitated ferroptosis by promoting autophagosome in pancreatic cancer cells

**Figure 6:**
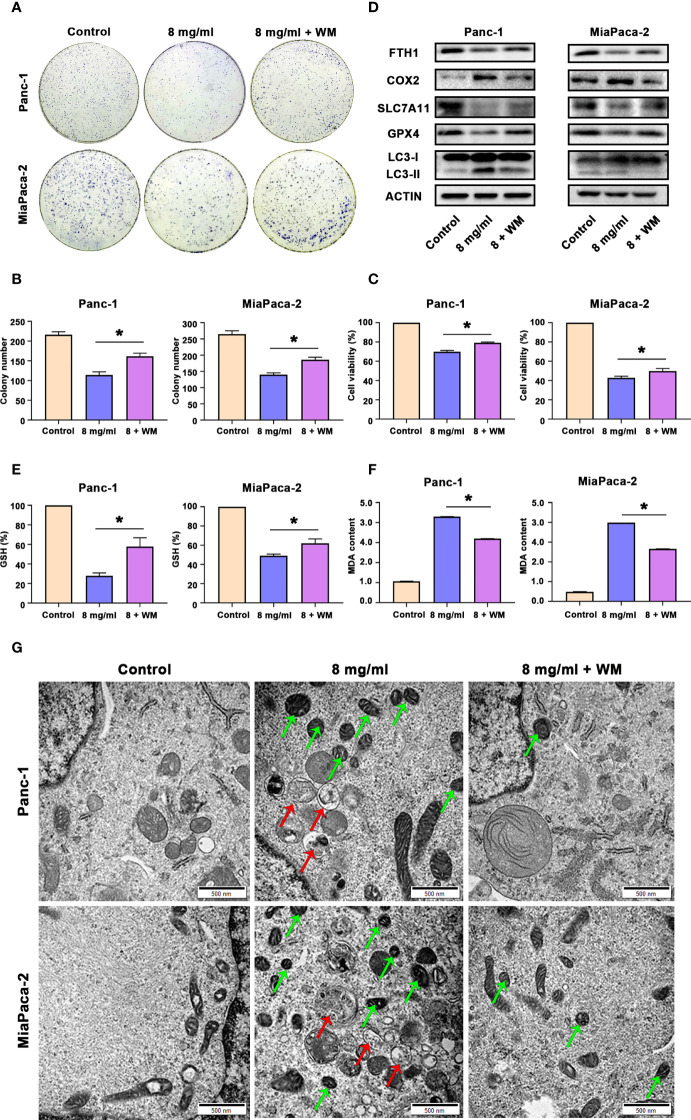
Huaier promoted ferroptosis by activating autophagy in pancreatic cancer cells. **(A, B)** Colony formation assay and its statistical analysis show cell proliferation of Panc-1 and MiaPaCa-2 cells after the control, Huaier and Huaier plus wortmannin (WM) treatments for 2 weeks. **(C)** CCK8 assay showing the viability of Panc-1 and MiaPaCa-2 cells after the control, Huaier and Huaier plus WM treatments for 96 h. **(D)** Western blotting showing the protein levels of FTH1, COX2, SLC7A11, GPX4 and LC3 in Panc-1 and MiaPaCa-2 cells treated with control, Huaier or Huaier plus WM. **(E, F)** The measurement of GSH and MDA concentrations in Panc-1 and MiaPaCa-2 cells after the control, Huaier and Huaier plus WM treatments. **(G)** TEM images showed ferroptosis-like mitochondria (green arrows) and autophagosomes (red arrows) in Panc-1 and MiaPaCa-2 cells after the control, Huaier and Huaier plus WM treatments. *P < 0.05, scale bar = 500 nm.

Since we proved above that Huaier can both increased autophagosome and induced ferroptosis in pancreatic cancer cells, and autophagy is also an inducer of ferroptosis in pancreatic cancer cells. Hence, we next investigated whether Huaier-induced ferroptosis was autophagy related. First, we pretreated cells with WM before exploring the cells under Huaier stress. CCK8 and colony formation assays showed that autophagosome inhibitor WM mitigated the cell viability/proliferation inhibition that was induced by Huaier (**Figures 6A–C**). These findings reviewed a fact that the promotion of autophagosomes induced by Huaier could contribute with ferroptosis and a possible interpretation could be that wortmannin, which prevent the biogenesis of autophagosomes, alleviates the phenotype. Notably, it has been demonstrated that the autophagic degradation (relies on smooth autophagy flux) of ferritin like ferritin heavy chain 1 (FTH1) leads to the accumulation of labile iron and promotes ferroptosis (22). Therefore, we measured the level of FTH1 and found the decrease of FTH1 protein by Huaier was reversed by WM (**Figure 6D**), which suggested that although there is no obvious promotion effect, Huaier did not inhibited the autophagy flux and consequently access accumulation of autophagosome. Supporting this view, different from the effect of Huaier on increasing the autophagosome and inducing ferroptosis, we found chloroquine (CQ), a classic autophagolysosome inhibitor (effect as the accumulation of autophagosomes and the blocking of autophagic flux), had no influence on the level of GSH and MDA (**Figure S1C, D**) and the decrease of FTH1 (data not showed), in other words, CQ have no effect on ferroptosis. Combined with above findings, we suggested that Huaier induced increasing of autophagosome may due to the promotion of its biogenesis primarily. And we also concluded that a smooth autophagy flux is required for the induction of ferroptosis again.

Then, we also discovered that the Huaier-induced downregulated expression of GPX4 and SLC7A11 and upregulated expression of COX2 and LC3-II were abolished by WM (**Figure 6D**). Indeed, WM restored the levels of GSH and reduced the content of MDA in Huaier-treated pancreatic cancer cells (**Figures 6E, F**). Additionally, the Prussian blue staining in **Figure S1E** showed that intracellular iron deposits increased in Huaier-treated pancreatic cancer cells, and this effect could be alleviated by WM. Most importantly, electron microscopy also indicated that Huaier treatment could induce autophagy (the red arrow reflects autophagosomes) and ferroptosis (the green arrow reflects peroxidation mitochondria), but these effects were suppressed by the autophagy inhibition induced by WM *in vitro* (**Figure 6G**). However, there was no difference between Huaier and Huaier plus Fer-1 in the LC3 level (labelled autophagy formation) *in vivo* (**Figure S1F**), which meant that Huaier-induced and autophagy-promoted ferroptosis may be a one-way process, but more detailed work is needed. Taken together, these results suggest that Huaier facilitated ferroptosis by promoting autophagosome in pancreatic cancer cells.

## Discussion

Pancreatic cancer has a very high mortality rate and there have been almost no obvious improvement in the 5-year survival rate over the past 10 years (1). It has become an major challenge for clinicians. Pancreatic cancer patients are mostly diagnosed at an advanced stage, so they often miss the opportunity for surgery; therefore, surgery plus adjuvant/neoadjuvant chemotherapy is the current mainstream treatments (23). Although chemo-strategies such as FOLFIRINOX and the combination of gemcitabine and S-1/capecitabine/albumin paclitaxel have been developed in recent years (24), the low sensitivity and high rate of PDAC resistance and its underlying mechanisms remain largely unresolved (25). Thus, investigation into the molecular mechanism of chemotherapy resistance and new chemotherapeutic drugs in pancreatic cancer is urgently needed to improve patient outcomes.

Mounting evidence has demonstrated that natural compounds may have great therapeutic potential for malignancies, and Huaier has been proven to have an anti-tumour effect in several cancer types, such as lung adenocarcinoma. Huaier induces apoptosis and inhibits the proliferation of cancer cells by modulating the miR-26b-5p-EZH2 axis (26). In cholangiocarcinoma, a homogeneous polysaccharide from the fruit bodies of Huaier, W-NTRP, has immunoregulatory and anti-tumour activities *in vitro (* 27). Additionally, in our previous research, we found that Huaier suppressed the proliferation of pancreatic cancer (11), but the mechanism remains unclear.

There are various mechanisms for cell death, including apoptosis, autophagy, necrosis, necroptosis, and ferroptosis (28). Next, we tried to determine how Huaier inhibits proliferation and induces cell death in PDAC. It has previously been reported that Huaier induces pancreatic cancer cell apoptosis (11); however, in the present study, no apoptotic bodies or other apoptotic morphology changes could be found after photographing Huaier-treated cells with an electron microscope. Interestingly, we found a meaningful phenomenon that was consistent with the description of ferroptosis. After the Huaier treatment, pancreatic cancer cells showed signs of mitochondrial volume reduction, membrane density increase and mitochondrial cristae fusion or disappearance (29). These findings lay the foundation for our subsequent experiments to study ferroptosis induction by Huaier in PDAC.

Ferroptosis is a novel form of non-apoptotic cell death that was first proposed by Dixon in 2012 (30). Intracellular depletion of GSH specifically triggers this form of cell death (31). In particular, the anti-tumour effect of ferroptosis has been researched in a wide spectrum of malignant tumours. Several studies indicate that ferroptosis may counteract tumour growth by regulating the suppressor gene p53, which prevents cell proliferation or induces cell death (31). In addition, the tumour-suppressive role of tumour suppressor BRCA1-associated protein 1 (BAP1) with ferroptosis has been validated in clear cell renal cell carcinoma (32). These findings provide new perspectives on ferroptosis for the treatment of cancer. To date, no studies have shown the link between ferroptosis and the anti-tumour effect of Huaier. To confirm that Huaier could induce ferroptosis, we designed experiments to explore ferroptosis markers. There are three key hallmarks of ferroptosis, including the impairment in repair capacity of lipid peroxide that is caused by the loss of GPX4 activity, the availability of redox-active iron, and the oxidation of polyunsaturated fatty acid (PUFA)-containing phospholipids (33). Therefore, in the following experiment, we measured the GSH and MDA contents after Huaier treatment because GPX4 and MDA could reflect oxidative stress (34). We also measured the changes in the ferroptosis-related proteins GPX4, SLC7A11 and COX2 after Huaier treatment. Overall, these results indicated that ferroptosis was induced by Huaier in pancreatic cancer. Notably, apoptosis should not be excluded in Huaier treatments, and ferroptosis induction is just one of the several mechanisms by which Huaier induces cancer cell death.

Next, we explored the mechanism by which Huaier induces ferroptosis. According to the most recent study, the excessive production of ROS is important to induce ferroptosis (17). Excessive levels of iron induce oxidative stress *via* Fenton and redox reactions, resulting in increased production of ROS (35). Therefore, we examined the effect of Huaier on ROS production and found that Huaier upregulated ROS levels in a concentration-dependent manner. In addition to dominating ferroptosis, ROS can also promote autophagy (17), a lysosome-dependent self-degradation process that exists widely in mammals. Interestingly, during the TEM observations, apart from the characteristics of ferroptosis, we also observed an obvious increase in autophagosomes after the Huaier treatment in PDAC cells. The immunofluorescence and western blotting results also suggested that Huaier promotes autophagy, especially on the promotion of autophagosomes. However, the ad-mRFP-GFP-LC3 system showed that the increase in autophagosomes caused by Huaier treatment cannot rule out the accumulation effect after inhibition of autophagolysosome formation, namely the blocking of autophagy flux. Hence, considering that the smooth autophagy flux can lead to the degradation (occur in autophagolysosome) of cellular ferritin such as FTH1 and thus cause an increase in cellular labile iron levels, which is the initiating event of ferroptosis (36, 37),we aim to explore whether Huaier can influence the level of FTH1, in other words, Huaier can blocked the autophagy flux or not. And the answer is no. The results showed an obvious reduction in FTH1 expression in the Huaier group, and this effect can be revised by an autophagosomes inhibitor WM. Combining the fact that Huaier can promote the EGFR/ERK pathway and the AMPK/ULK axis and that CQ have no effect on ferroptosis, we believe that Huaier mainly induces the increase of autophagosomes by promoting autophagosome formation, rather than blocking autophagic flux, which eventually leads to the accumulation of autophagosomes. Hence, in summary, all the findings suggest that autophagy may be the bridge between Huaier and ferroptosis. Consistent with this, we found a profound decrease in ferroptosis induced by Huaier in the background of WM. A cell viability CCK8 assay and colony formation analysis also confirmed that the inhibition of autophagy can decrease the anti-tumour effects of Huaier. These data may illustrate that Huaier may stimulate autophagic degradation of ferritin and increase cellular labile iron, thus promoting the death of pancreatic cancer cells.

Besides of this, there are still other limitations in our study. First, we suggested that Huaier could induce the death of pancreatic cancer cells. The death mechanism is ferroptosis and is accompanied by ROS accumulation and autophagy upregulation. Although we did not observe signs of apoptosis in the TEM morphology, Huaier-induced apoptosis should not be excluded in the Huaier anti-tumour mechanism. The relationship between apoptosis and ferroptosis in Huaier treatment was not explored in the present study since we focused on ferroptosis. Further efforts can be made by interested researchers. Second, we confirmed ROS accumulation, autophagosome upregulation and ferroptosis formation in Huaier-treated pancreatic cancer cells, but we did not fully investigate the mechanisms between ROS-autophagy and how autophagy precisely induced ferroptosis in the background of Huaier. These questions could also be a good direction of study in the future. Third, Huaier is now usually used for patients that are undergoing gemcitabine treatments. We explored the single usage of Huaier *in vivo* and *in vitro* but did not perform the combination assay of Huaier and gemcitabine. Therefore, whether ferroptosis induction exists and how much the ferroptosis induced by Huaier contributes to anti-tumour effects have not been elucidated clearly in the combined use of gemcitabine and Huaier. In addition, in PDAC tissue, cancer cells are not the only cell type. Mesenchymal cells and the ECM are also important in PDAC. We explored the effect of Huaier on cancer cells, but its role on mesenchymal cells, such as pancreatic stellate cells, was not explored, and whether Huaier could induce ferroptosis in normal pancreatic ductal cell or whether the induction was specific to cancer cells was not explored. Finally, since Huaier is a compound (41.5% polysaccharides, 12.93% amino acids and 8.72% water) and cannot be separated into specific molecules, we cannot identify the specific molecule that functions in ferroptosis. Techniques in chemistry are needed to improve this issue and identify the reactive components in Huaier extracts.

## Conclusion

Overall, we investigated a new anti-tumour mechanism for Huaier by inducing cancer cell ferroptosis both *in vivo* and *in vitro*. ROS accumulation and abnormal autophagy-related ferritin degradation are potential mechanisms by which Huaier induces ferroptosis. Our experiments enable to promote understanding of the tumour biology and the working mechanism of Huaier in anti-tumour treatments. It may be a new direction for the utilisation of next-generation therapy for PDAC.

## Data availability statement

The original contributions presented in the study are included in the article/**Supplementary Material**. Further inquiries can be directed to the corresponding authors.

## Ethics statement

The animal study was reviewed and approved by Animal Care Committee of First Affiliated Hospital of Xi’an Jiaotong University, Xi’an, China.

## Author contributions

WQ and ZWa designed the experiments; ZZ carried out most of the experiments with the guidance of WQ, WZ, MG, SZ, BY, and BQ analysed the data and organised the figures; ZZ wrote the manuscript, XW, ZWu, and WQ modified it during the original stage and the review stage. They were helped by ZWu and QM. All authors read and approved the final manuscript.

## Funding

This study was funded by the National Natural Science Foundation of China (No. 82072702, 82103117, 81872008, 82103563 and 82172853). This study was also supported by the Science and Technology Innovation Team Program of Shaanxi province (No. 2020SF231), the Clinical Research Award of the First Affiliated Hospital of Xi’an Jiaotong University (No. XJTU1AF-CRF-2019-005), and Project supported by the Natural ScienceFoundation of Shaanxi Province, China (Grant No.2020JQ-510 and2020SF-231).

## Conflict of interest

The authors declare that the research was conducted in the absence of any commercial or financial relationships that could be construed as a potential conflict of interest.

## Publisher’s note

All claims expressed in this article are solely those of the authors and do not necessarily represent those of their affiliated organizations, or those of the publisher, the editors and the reviewers. Any product that may be evaluated in this article, or claim that may be made by its manufacturer, is not guaranteed or endorsed by the publisher.

## Supplementary material

Supplementary Figure 1 Prussian blue staining and partial IHC staining among the different treatment groups in this study. **(A)** Prussian blue staining showing the iron accumulation (blue dots and green arrows) in Panc-1 and MiaPaCa-2 cells after Huaier treatment. **(B)** Prussian blue staining showing the iron accumulation (blue dots and green arrows) in Panc-1 and MiaPaCa-2 cells after the control, RSL3, RSL3 plus WM and RSL3 plus RA treatments. **(C, D)** The measurement of GSH and MDA concentrations in Panc-1 and MiaPaCa-2 cells after the control, chloroquine (CQ) and RSL3 treatments. **(E)** Prussian blue staining showing the iron accumulation (blue dots and green arrows) in Panc-1 and MiaPaCa-2 cells after the control, Huaier and Huaier plus WM treatments. **(F)** IHC staining of LC3 showing the autophagy status of pancreatic cancer tumours in the control, Huaier and Huaier plus Fer-1 groups. ns P > 0.05, *P < 0.05, scale bar = 100 μm.
