# Supplementary figures and images for: Huaier suppresses pancreatic cancer progression via activating cell autophagy induced ferroptosis
Source: Front Oncol. 2022 Sep 30;12:960858. doi: 10.3389/fonc.2022.960858 (PMC9561879; doi:10.3389/fonc.2022.960858)

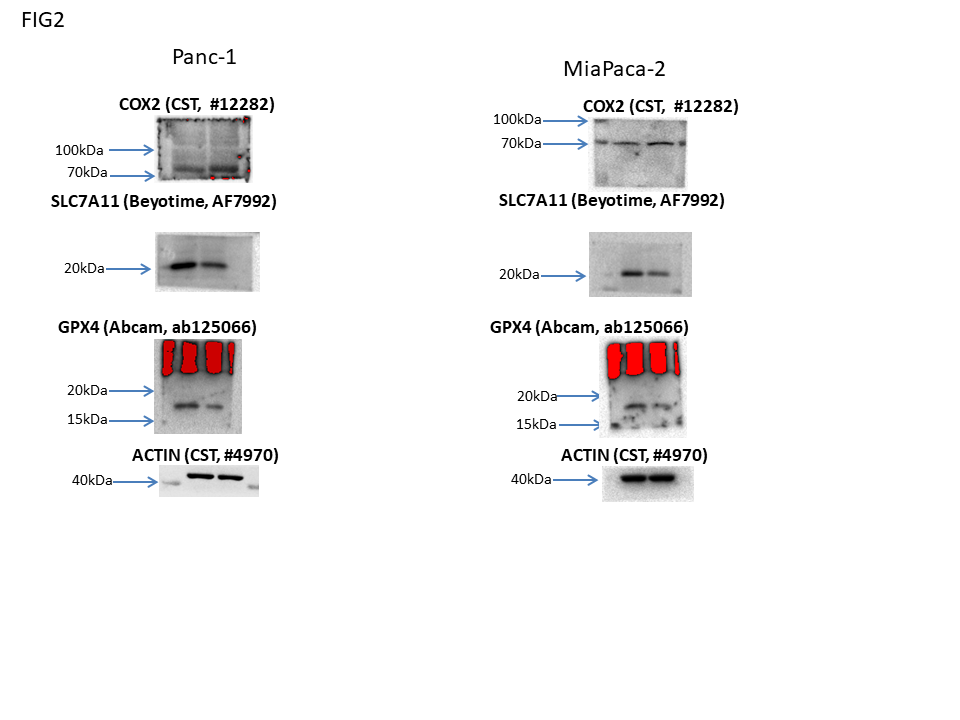

Supplement: Supplementary file 2 [file DataSheet_1.zip › WB/幻灯片1.TIF]

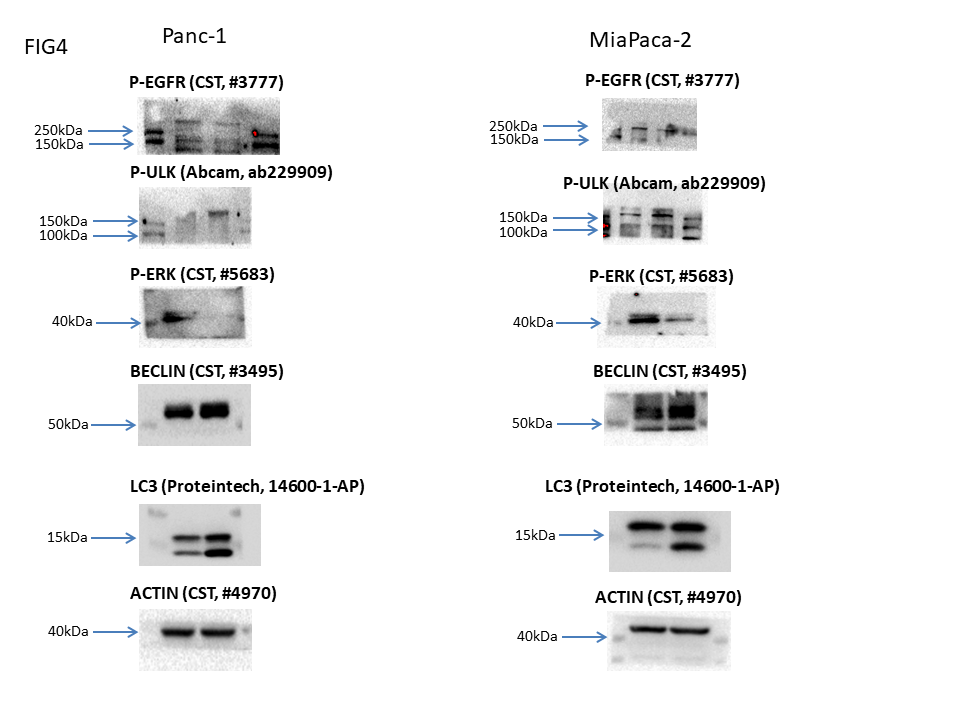

Supplement: Supplementary file 2 [file DataSheet_1.zip › WB/幻灯片2.TIF]

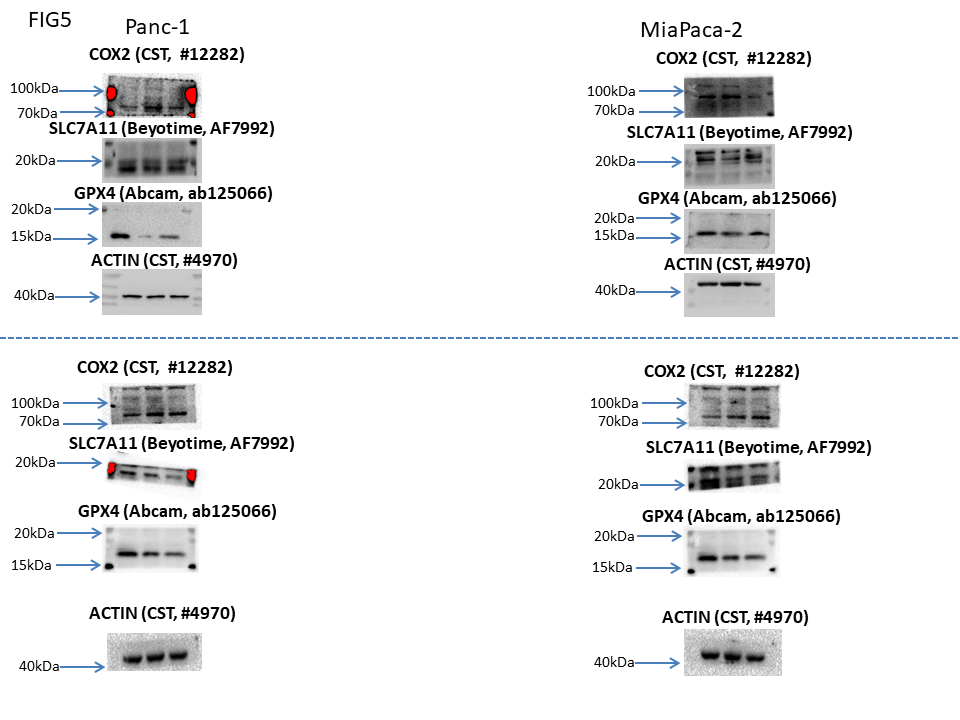

Supplement: Supplementary file 2 [file DataSheet_1.zip › WB/幻灯片3.TIF]

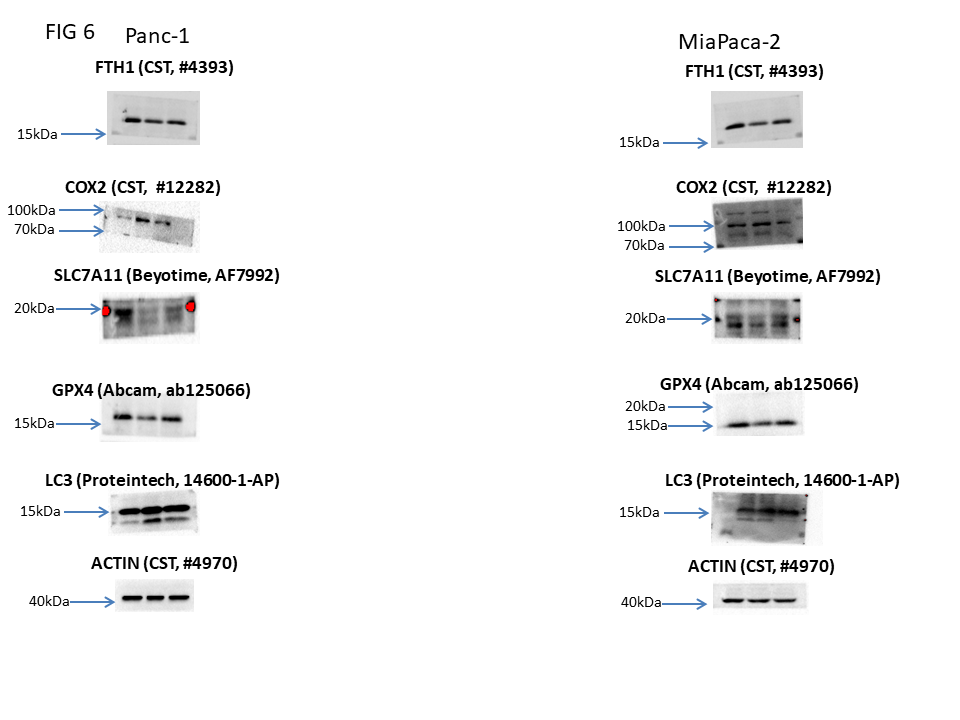

Supplement: Supplementary file 2 [file DataSheet_1.zip › WB/幻灯片4.TIF]

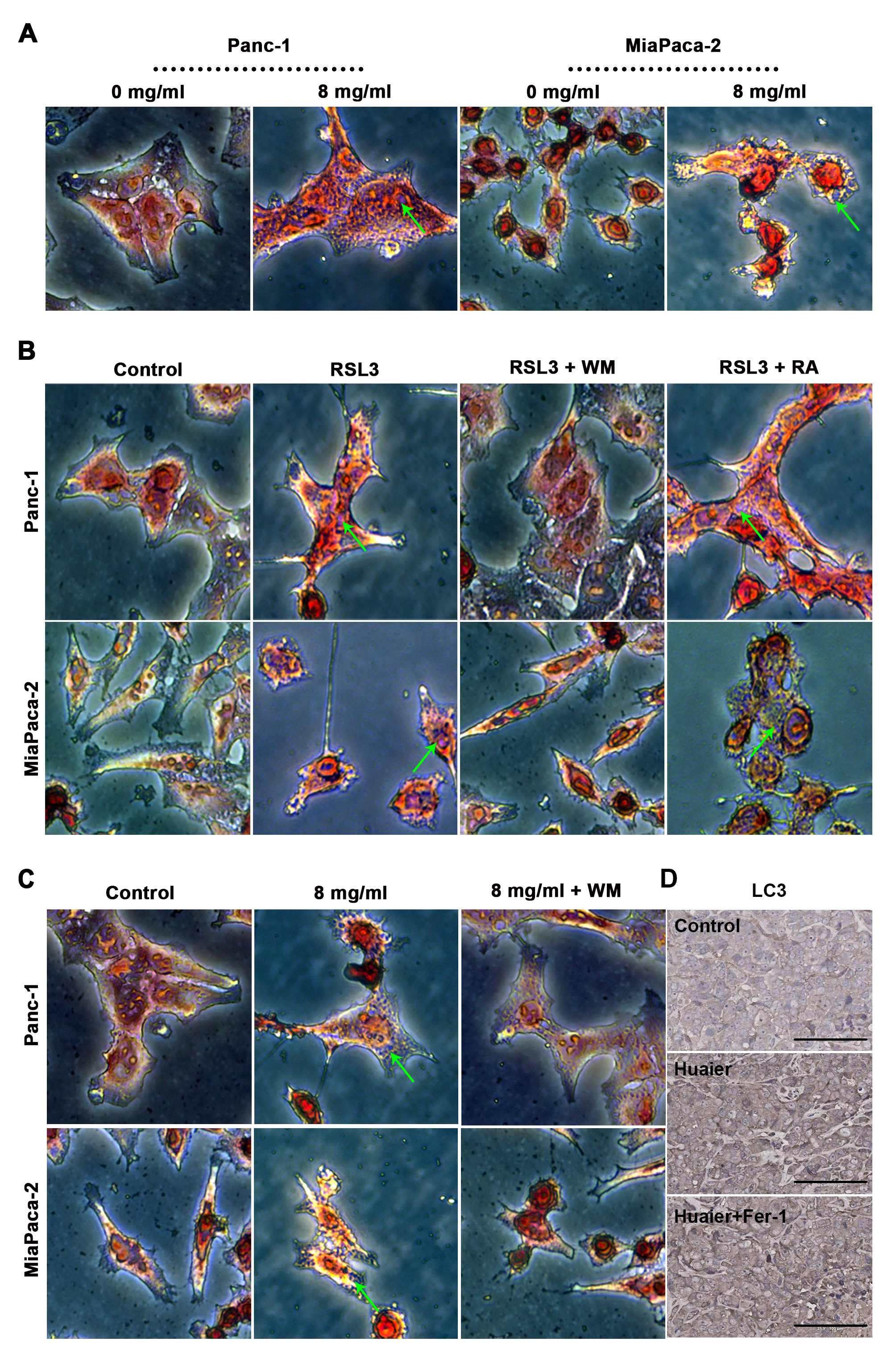

Supplement: Supplementary Figure 1 — Prussian blue staining and partial IHC staining among the different treatment groups in this study. (A) Prussian blue staining showing the iron accumulation (blue dots and green arrows) in Panc-1 and MiaPaCa-2 cells after Huaier treatment. (B) Prussian blue staining showing the iron accumulation (blue dots and green arrows) in Panc-1 and MiaPaCa-2 cells after the control, RSL3, RSL3 plus WM and RSL3 plus RA treatments. (C, D) The measurement of GSH and MDA concentrations in Panc-1 and MiaPaCa-2 cells after the control, chloroquine (CQ) and RSL3 treatments. (E) Prussian blue staining showing the iron accumulation (blue dots and green arrows) in Panc-1 and MiaPaCa-2 cells after the control, Huaier and Huaier plus WM treatments. (F) IHC staining of LC3 showing the autophagy status of pancreatic cancer tumours in the control, Huaier and Huaier plus Fer-1 groups. ns P > 0.05, *P < 0.05, scale bar = 100 μm. [file Image_1.tif]
